# Pilot testing of dipsticks as point-of-care assays for rapid diagnosis of poor-quality artemisinin drugs in endemic settings

**DOI:** 10.1186/s41182-016-0015-8

**Published:** 2016-05-16

**Authors:** Suqin Guo, Lishan He, Daniel J. Tisch, James Kazura, Sungano Mharakurwa, Jagadish Mahanta, Sócrates Herrera, Baomin Wang, Liwang Cui

**Affiliations:** College of Agronomy and Biotechnology, China Agricultural University, Beijing, China; Department of Epidemiology and Biostatistics, Case Western Reserve University, Cleveland, OH USA; Center for Global Health and Diseases, Case Western Reserve University, Cleveland, OH USA; Malaria Research Department, Macha Research Trust, Johns Hopkins Malaria Research Institute, Choma, Zambia; Regional Medical Research Centre (NE), Dibrugarh, 786001 Assam India; Caucaseco Scientific Research Center and Malaria Vaccine and Drug Development Center, Cali, Colombia; Department of Entomology, Pennsylvania State University, University Park, PA 16802 USA; Present address: College of Agronomy, Guangxi University, 530004 Nanning, China

**Keywords:** Artemisinin-based combination therapies (ACTs), Dipstick, Malaria, Antibody

## Abstract

**Background:**

Good-quality artemisinin drugs are essential for malaria treatment, but increasing prevalence of poor-quality artemisinin drugs in many endemic countries hinders effective management of malaria cases.

**Methods:**

To develop a point-of-care assay for rapid identification of counterfeit and substandard artemisinin drugs for resource-limited areas, we used specific monoclonal antibodies against artesunate and artemether, and developed prototypes of lateral flow dipstick assays. In this pilot test, we evaluated the feasibility of these dipsticks under different endemic settings and their performance in the hands of untrained personnel.

**Results:**

The results showed that the dipstick tests can be successfully performed by different investigators with the included instruction sheet. None of the artemether and artesunate drugs collected from public pharmacies in different endemic countries failed the test.

**Conclusion:**

It is possible that the simple dipstick assays, with future optimization of test conditions and sensitivity, can be used as a qualitative and semi-quantitative assay for rapid screening of counterfeit artemisinin drugs in endemic settings.

## Background

It is estimated that 10–30 % of the pharmaceuticals in the world are of poor quality [1]. Generally, poor-quality drugs are falsified (deliberately and fraudulently manufactured and mislabeled), substandard (produced by manufacturers authorized by regulatory authorities, but not able to meet quality specifications), or degraded during transport and storage. Low-income countries with a high malaria burden are particular targets for falsified drugs. The high disease burden in these countries and resultant wide use of drugs make these areas lucrative locations for the counterfeit drug business. Artemisinin-based combination therapies (ACTs), as the recommended first-line treatments for uncomplicated *Plasmodium falciparum* malaria, have played an important role in reducing the global malaria-associated mortality and morbidity. Good-quality ACTs are essential for delivering effective malaria case management, but the increasing proportions of poor-quality (both counterfeit and substandard) drugs in many endemic countries are concerning [2, 3]. Counterfeit artemisinins containing little or no active ingredient provide inadequate treatment and can be life-threatening [4]. In Sub-Saharan Africa, it has been estimated that a large number of deaths of children under age 5 were associated with consumption of poor-quality antimalarials [5]. Therefore, heightened surveillance and regulatory efforts are urgently needed for combating the entry and circulation of poor-quality antimalarial drugs.

Accurate determination of artemisinin contents in commercial drugs requires sophisticated instrumentation and expertise. In resource-limited settings, thin-layer chromatography (TLC) is the often-used method for pharmaceutical analysis. For example, TLC, implemented in the Minilab from Global Pharma Health Fund, is a system recommended by the World Health Organization (WHO) for the detection of fake drugs. This method, however, is qualitative, cumbersome, and laborious, and it requires trained personnel to implement. In response to the counterfeit artesunate monotherapy tablets in Southeast Asia, a colorimetric method was developed for rapidly assessing drug authenticity [6, 7]. This method uses the Fast Red TR salt to produce a yellow color upon reaction with artemisinin derivatives, but this test cannot be used in ACTs containing a yellow-colored partner drug such as lumefantrine and amodiaquine. Recently, a new colorimetric method was developed, but it requires caustic acids in the assay, which can be difficult to manage in remote endemic settings [8]. To address the current limitations of point-of-care (POC) diagnoses of fake artemisinins, we developed a series of monoclonal antibodies (mAbs) that showed different specificities to artemisinin derivatives [9, 10] for potential use in immunoassays for artemisinin quantitation. Given that workers in malaria-endemic populations are very familiar with the rapid diagnostic test (RDT) format for malaria diagnosis [11], we chose to develop a lateral flow dipstick for POC tests that would provide qualitative and semi-quantitative detection of artemisinins in antimalarial drugs [12]. A prototype dipstick was designed based on one selected mAb that was found to have high avidity and broad reactivity for artemisinins, with sensitivity as low as 100–200 and 200–500 ng/ml for artesunate and dihydroartemisinin, respectively [12]. Recently, we have developed additional mAbs that are highly specific for artemether and artesunate, respectively [10, 13], and we have attempted to improve the dipsticks and make them specific for each of the artemisinin derivatives commonly found in ACT drugs. To further improve these dipsticks and to determine how these dipstick tests perform under field settings, we distributed some of the tests to several International Centers of Excellence for Malaria Research (ICEMRs) for pilot evaluations. Here, we report the results from these field evaluations and make recommendations for improvements of the tests.

## Methods

### Development of colloidal gold-based lateral-flow dipsticks

**Fig. 1:**
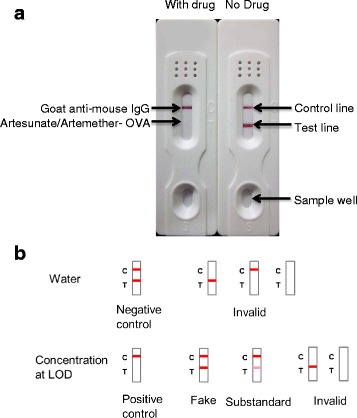
**a** The assembled dipsticks for analysis of artesunate/artemether-containing drugs. The disappearance of the test line indicates that concentration of the tested drug is above the limit of detection of the dipstick. **b** Description of the test results according to the instruction sheet. Concentration at LOD (limit of detection) shows the potential results of the tested drug when diluted to the LOD based on the described drug content

We have developed a dipstick that can be used as a POC test for artemisinin-, artesunate-, and dihydroartemisinin-containing drugs using a mAb (3D_8_2G_7_) that has considerable cross-reactivities to these three compounds [12]. To differentiate artemisinin and its derivatives in different ACT drugs, we developed a dipstick using a mAb (3D_8_2G_6_) that is specific for artesunate [13]. Recently, based on a mAb (2G12E1) that is specific for artemether [10], we developed a dipstick specifically for detecting artemether in ACT drugs (described elsewhere). Here, we used mAb 2G12E1 and mAb 3D_8_2G_6_ to prepare two types of dipsticks that are specific for artemether and artesunate, respectively. Preparation of colloidal gold-conjugated antibodies and assembly of the dipsticks were performed as described earlier [12]. The sensitivities of these batches of dipsticks (indicator ranges) were determined using serially diluted artemether and artesunate, respectively [12]. The final dipsticks (Fig. 1a) were then sealed in a plastic case with desiccant gel and, together with the instruction sheet, sent to different laboratories for evaluation.

### Field testing of the dipsticks

Different brands of artemether- and artesunate-containing drugs (19 artemether-lumefantrine drugs, 4 artesunate injections, and 1 artesunate-pyrimethamine-sulphadoxine drug) were purchased from local pharmacies in Colombia, India, Papua New Guinea (PNG), and Zambia. None of the drugs had passed their expiration dates at the time of test. All tests were conducted locally under field conditions following the instructions on the instruction sheet. The instruction sheet included with the dipsticks described the type of dipstick included, the materials provided (dipstick and a dropper), sample extraction and dilution schemes, steps for reading the dipstick, and instructions for interpreting the results, all of which are detailed below.

### Sample extraction

Individual drug tablets were crushed in a piece of folded paper to a fine powder, which was transferred to ≥95 % alcohol to produce a content of 2 mg/ml based on the labeled content of the commercial drug. For artesunate injections, the drug powders were dissolved similarly in ≥95 % alcohol.

### Dilution

Using a dropper included in the assay, a drop of the stock was transferred to a cup containing 1.25 ml of water for artesunate or 12.5 ml for artemether, which should give a theoretical concentration of the drug of ~40 μg/ml artesunate or ~4 μg/ml of artemether. These different dilution schemes were used given that the two dipsticks had different indicator ranges.

### Dipstick assay

After rinsing the dropper at least three times with water, the dropper was used to add three drops (or 80 μl) of the diluted drugs to the sample well of the dipstick. The color of the control and/or test lines was to be visible in 5 min (not beyond 15 min).

### Result interpretation

For a valid assay with the control line visible, if the test line showed no color, the drug was to be considered qualified (Fig. 1b). If the test line showed a dark or faint color, it suggested that the drug might be fake or substandard. We suggested that the drug needed to be tested at a drug concentration ten times higher (ten times less dilution) to confirm the finding.

## Results

The two mAbs against artemether and artesunate were highly specific for these two artemisinin derivatives, respectively. They were used to develop highly sensitive, indirect competitive enzyme-linked immunosorbent assays for accurate determination of artemether and artesunate concentrations [10]. Since dipstick assays are used to measure high concentrations of drugs, we specifically lowered the sensitivity of the assay to avoid multiple dilution steps. Using serially diluted standard drugs, the indicator ranges of the artemether and artesunate dipsticks were determined in the laboratory as 4–8 and 40–50 μg/ml, respectively.

**Fig. 2:**
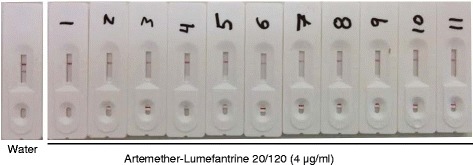
Artemether/lumefantrine (20/120) samples tested in PNG showing that all tested drugs passed the tests. Drugs tested include Artefan (1–3 and 5–8), Lumartem (4), Coartem (9 and 10), and Coatal (11). The order of the dipsticks shown here is the same as the order listed in Table 1 for the artemether/lumefantrine drugs tested in PNG

Since the primary goal of this pilot field test was to investigate the performances of the dipsticks as POC devices in endemic settings, we wanted to know the performances of the dipsticks in the hands of different investigators under field conditions. Given the simplicity of the dipstick assay and the familiarity of most malaria-endemic populations with the RDT format, no specific training on the use of the assay was offered. All investigators followed the instructions and successfully performed the dipstick assays under field conditions. After initially dissolving the drug powder in ≥95 % alcohol, the stock solutions were further diluted using regular bottled water. Since the main purpose was to test applicability of the dipsticks in remote endemic settings, rather than to screen counterfeit and substandard drugs, all drugs were purchased from pharmacies in public clinics and hospitals. The test results showed that all tested artemether/lumefantrine, artesunate/pyrimethamine/sulphadoxine, and artesunate injections contained the described amounts of active pharmaceutical ingredients (Fig. 2, Table 1).

**Table 1:** Test results of ACT commercial drugs and artesunate injections by dipsticks in PNG, India, and Zambia

Drug name^a^	Drug composition	Area of procurement	Result
Larinate-200 Kit	Artesunate 200 mg pyrimethamine 25 mg sulphadoxine 500 mg	Agartala, Tripura, India	Qualified
Lumether Forte DT	Artemether 80 mg lumefantrine 480 mg	Agartala, Tripura, India	Qualified
Lumerax-80	Artemether 80 mg lumefantrine 480 mg	Agartala, Tripura, India	Qualified
Micromether-LF	Artemether 80 mg lumefantrine 480 mg	Tinsukia, Assam, India	Qualified
Falcigo	Artesunate 60 mg	Dibrugarh, Assam, India	Qualified
Rtsunate	Artesunate 60 mg	Dibrugarh, Assam, India	Qualified
Artesun—60 mg vial for IM/IV	Artesunate 60 mg	Mugil Clinic, Madang, PNG	Qualified
Artesun—60 mg vial for IM/IV	Artesunate 60 mg	Mugil Clinic, Madang, PNG	Qualified
Artefan 20/120—packaged for 15–24 kg	Artemether 20 mg lumefantrine 120 mg	Elaita Clinic, Maprik, PNG	Qualified
Artefan 20/120—packaged for 15–24 kg	Artemether 20 mg lumefantrine 120 mg	Elaita Clinic, Maprik, PNG	Qualified
Artefan 20/120—packaged for >35 kg and adults	Artemether 20 mg lumefantrine 120 mg	Mugil Clinic, Madang, PNG	Qualified
Lumartem—packaged for 5–15 kg	Artemether 20 mg lumefantrine 120 mg	Mugil Clinic, Madang, PNG	Qualified
Artefan 20/120—packaged for 15–24 kg	Artemether 20 mg lumefantrine 120 mg	Mugil Clinic, Madang, PNG	Qualified
Artefan 20/120—packaged for >35 kg and adults	Artemether 20 mg lumefantrine 120 mg	Maprik Hospital, PNG	Qualified
Artefan 20/120—packaged for 25–34 kg	Artemether 20 mg lumefantrine 120 mg	Elaita Clinic, Maprik, PNG	Qualified
Artefan 20/120—packaged for 15–24 kg	Artemether 20 mg lumefantrine 120 mg	Elaita Clinic, Maprik, PNG	Qualified
Coartem 20/120—packaged for 15–24 kg	Artemether 20 mg lumefantrine 120 mg	Elaita Clinic, Maprik, PNG	Qualified
Coartem 20/120—packaged for 15–24 kg	Artemether 20 mg lumefantrine 120 mg	PNGIMR Maprik, PNG	Qualified
Coatal 20/120—packaged with dosage chart	Artemether 20 mg lumefantrine 120 mg	Wewak Pharmacy, PNG	Qualified
Coartem 20/120 (5 different lots)	Artemether 20 mg lumefantrine 120 mg	Zambia	Qualified

^a^Each sample was analyzed in triplicate

Additional evaluations using slight variations of the protocol also were performed in different field sites. In PNG, when initial drugs (artemether 20 mg/lumefantrine 120 mg) were dissolved in 10 ml of water as stock solutions, diluted in water to ~2 μg/ml, and tested on the artemether dipstick, the test line in the dipstick showed a faint band compared to the complete disappearance that occurred when the same drugs were dissolved in alcohol per instruction. This result is consistent with the poor solubility of artemether in water. At the Colombian site (Buernaventura, Valle), investigators also applied the same CoArtem® 20/120 drugs on both types of dipsticks and found that the artemether dipstick produced a result showing the drug was qualified, whereas the same drug failed the artesunate dipstick. This is consistent with the high specificities and low cross-reactivities of the two mAbs used to develop the two dipsticks. At the Indian site (Assam State), investigators also tried six dilutions of artesunate with final concentrations of 2, 4, 8, 10, 20, and 40 μg/ml on the artesunate dipsticks. The result showed that the test line was clearly visible at 2 μg/ml, faint at 4 μg/ml, and completely invisible at 8 μg/ml, which agreed well with the sensitivity of the dipstick determined under laboratory conditions.

## Discussion

We have demonstrated that the mAb-based dipstick assays for detecting artemisinin and its derivatives in ACT drugs performed well in field conditions without the requirement for technical training of the test performers. Compared to most of the currently available assays for quality control of artemisinin-containing drugs at field sites, this method offers several advantages. First, it has a very simple sample preparation step, which involves crushing drug tablets, dissolving the drug powder in ≥95 % alcohol, and further diluting the solution with water. Second, the protocol is fast and takes about 10 min from the beginning of the assay to the final reading of the result. Third, we have found that ≥95 % alcohol for the initial preparation of the stock solution performed sufficiently well for field-testing purposes, thus avoiding the need for flammable organic solvents, such as acetonitrile. Fourth, as shown in the lab tests, the partner drugs in the ACTs do not interfere with the assays, though more vigorous tests are needed on other ACTs such as artesunate/mefloquine and artesunate/amodiaquine. Finally, the high specificities of these two mAbs for artemether and artesunate with only 2–4 % cross-reactivity to artemisinin give the dipsticks another advantage for identifying ACTs containing these ingredients only [10, 13]. Potentially, these dipsticks could identify counterfeit drugs containing the easily obtainable natural product artemisinin, which may thwart some chemical tests. These features make the dipstick assay a truly suitable POC device for preliminary quality screening of artemisinin drugs in resource-limiting countries.

In recognition of the challenges for detecting counterfeit antimalarials in developing countries, Green et al. proposed a three-tiered drug evaluation strategy [8]. We envision that the dipstick device could be a nice fit as a Tier III assay for the purpose of screening to identify suspicious counterfeit drugs since the amount of the active ingredients can be semi-quantitatively assessed. Dipsticks can easily be carried to highly remote areas and used on site, while the results can be sent back to regulatory agencies as digital cell phone pictures. Suspicious drugs can then be sent to central labs for accurate testing. Furthermore, each batch of dipsticks has a roughly defined sensitivity range, thus allowing the performers to obtain an estimated range of the drug content. Moreover, by performing different dilutions of the sample, the drug content can be more narrowly defined. Therefore, the dipsticks also allow semi-quantitative analysis of artemisinins in the ACTs. The current dipsticks had been stored at 4 °C for over 2 months and under ambient temperature for about 2 weeks. No significant reduction in sensitivity was detected. Their shelf life, especially under tropical climate, needs to be vigorously tested.

Further evaluations and improvements of the dipsticks are needed. Since different dipsticks are designed for different ACTs, the specificity needs to be marked clearly on the tests to avoid confusion and misuse, since different ACTs are often available in the same regions. The poor water solubility of artemether makes the use of ≥95 % alcohol necessary. In recognition of the potential difficulty in obtaining ≥95 % alcohol in endemic settings, we have tried to produce the artesunate dipsticks with a lower detection limit (8 μg/ml). Optimization of the dipsticks may allow the drugs to be tested at much higher dilutions directly in water. Further vigorous tests are needed to determine the stability of the dipsticks under endemic conditions.

## Conclusions

Dipsticks based on specific mAbs against artemisinin derivatives have been developed as POC assays for rapid quality control of ACTs in endemic settings. Field evaluations by different investigators confirmed the satisfactory performance of and applicability of the tests to artesunate- and artemether-containing drugs. Though the results are qualitative and maximally semi-quantitative, these simple assays are well suited for a quick screen to identify suspicious fake and substandard artemisinin-containing drugs.

## References

[1] Newton PN, White NJ, Rozendaal JA, Green MD (2002). Murder by fake drugs. BMJ.

[2] Nayyar GM, Breman JG, Newton PN, Herrington J (2012). Poor-quality antimalarial drugs in Southeast Asia and sub-Saharan Africa. Lancet Infect Dis.

[3] Karunamoorthi K (2014). The counterfeit anti-malarial is a crime against humanity: a systematic review of the scientific evidence. Malar J.

[4] Newton PN, McGready R, Fernandez F, Green MD, Sunjio M, Bruneton C, Phanouvong S, Millet P, Whitty CJ, Talisuna AO (2006). Manslaughter by fake artesunate in Asia—will Africa be next?. PLoS Med.

[5] Renschler JP, Walters KM, Newton PN, Laxminarayan R (2015). Estimated under-five deaths associated with poor-quality antimalarials in sub-Saharan Africa. Am J Trop Med Hyg.

[6] Green MD, Mount DL, Wirtz RA (2001). Authentication of artemether, artesunate and dihydroartemisinin antimalarial tablets using a simple colorimetric method. Trop Med Int Health.

[7] Green MD, Mount DL, Wirtz RA, White NJ (2000). A colorimetric field method to assess the authenticity of drugs sold as the antimalarial artesunate. J Pharm Biomed Anal.

[8] Green MD, Hostetler DM, Nettey H, Swamidoss I, Ranieri N, Newton PN (2015). Integration of novel low-cost colorimetric, laser photometric, and visual fluorescent techniques for rapid identification of falsified medicines in resource-poor areas: application to artemether-lumefantrine. Am J Trop Med Hyg.

[9] Wang M, Cui Y, Zhou G, Yan G, Cui L, Wang B (2013). Validation of ELISA for quantitation of artemisinin-based antimalarial drugs. Am J Trop Med Hyg.

[10] Guo S, Cui Y, He L, Zhang L, Cao Z, Zhang W, Zhang R, Tan G, Wang B, Cui L (2013). Development of a specific monoclonal antibody-based ELISA to measure the artemether content of antimalarial drugs. PLoS One.

[11] Kobayashi T, Gamboa D, Ndiaye D, Cui L, Sutton PL, Vinetz JM (2015). Malaria diagnosis across the International Centers of Excellence for Malaria Research: platforms, performance, and standardization. Am J Trop Med Hyg.

[12] He L, Nan T, Cui Y, Guo S, Zhang W, Zhang R, Tan G, Wang B, Cui L (2014). Development of a colloidal gold-based lateral flow dipstick immunoassay for rapid qualitative and semi-quantitative analysis of artesunate and dihydroartemisinin. Malar J.

[13] Guo S, Zhang W, He L, Tan G, Min M, Kyaw MP, Wang B, Cui L: Rapid evaluation of artesunate quality with a specific monoclonal antibody-based lateral flow dipstick. *Anal Bioanal Chem* 2016. 10.1007/s00216-016-9363-9 PMC4982846 26873200

